# Surface-Enhanced Raman Spectroscopy Semi-Quantitative Molecular Profiling with a Convolutional Neural Network

**DOI:** 10.1177/00037028251377474

**Published:** 2025-08-31

**Authors:** Alexis Lebrun, Flavie Lavoie-Cardinal, Denis Boudreau

**Affiliations:** 1 33499 Centre d'optique, Photonique et Laser (COPL), Université Laval, Quebec, Canada; 2 Centre de Recherche CERVO, Université Laval, Quebec, Canada; 3 Institut Intelligence et Données, Université Laval, Quebec, Canada; 4 Département de Psychiatrie et Neurosciences, Université Laval, Quebec, Canada; 5 Département de Chimie, 12369 Université Laval, Quebec, Canada

**Keywords:** Deep learning, spectral classification, surface-enhanced Raman scattering, SERS, convolutional neural network, CNN

## Abstract

Surface-enhanced Raman scattering (SERS) spectroscopy represents a powerful analytical platform that combines non-destructive, label-free molecular identification with exceptional sensitivity for trace-level detection. Its capacity to generate information-rich spectral fingerprints makes SERS particularly advantageous for simultaneous multi-analyte analysis across diverse sample matrices, including complex biological systems. This study addresses the analytical challenges associated with identifying and quantifying multiple molecular species in complex environments by integrating SERS with advanced machine learning methodologies. We developed a hierarchical analytical framework that leverages the complementary strengths of deep learning and regression techniques: A multi-label convolutional neural network (CNN) for discriminating structurally similar analytes from SERS spectral data, coupled with a support vector regression (SVR) model for semi-quantitative determination of relative concentration ratios among identified species. The methodology was systematically validated using binary mixtures of short-chain fatty acids (SCFAs) as representative biomolecular targets, with performance rigorously benchmarked against established multivariate statistical methods and conventional machine learning approaches. Experimental validation demonstrated robust classification accuracy for both analytes at physiologically relevant concentrations, maintaining consistent performance across simple aqueous media and complex cell culture environments. These results establish the viability of the integrated SERS-CNN-SVR approach for advanced mixture analysis applications where precise identification and quantification of multiple biomarkers is essential.

## Graphical abstract

**Graphical abstract:**
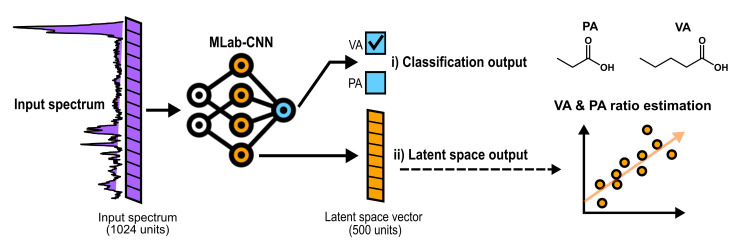
This is a visual representation of the abstract.

## Introduction

Surface-enhanced Raman spectroscopy (SERS) has attracted growing interest over the years across a wide range of applications, such as environmental monitoring,^1–3^ biomedicine,^4,5^ and electrochemistry,^6^ among others, in part due to the ability to produce information-rich spectra on the molecular composition of a sample, featuring distinct spectral bands that can be assigned to particular functional groups. Moreover, compared to conventional Raman spectroscopy, SERS achieves significant higher sensitivity when analyte molecules are exposed to the strong electromagnetic field induced at the surface of metal nanostructures by a resonant incident light source, i.e., localized surface plasmon resonance (LSPR),^7,8^ allowing the detection of molecular species at very low concentrations, even at single-molecule level.^9–11^ This label-free and non-invasive technique represents a compelling alternative to conventional instrumental analysis methods across numerous applications, particularly in clinical and biomedical diagnostics.^12^ The spectral richness inherent to SERS data make it particularly well suited to the simultaneous multiplexed detection of biomarkers and pathogens found within diverse biological matrices.^13,14^ For example, SERS spectroscopy was recently used to identify protein biomarkers expressed in breast cancer cells,^15^ analyze serum metabolic profiles induced by SARS-CoV-2,^16^ and detect and diagnose medical conditions such as diabetes,^17^ chronic kidney disease (CKD),^18^ and Crohn’s disease.^19^

Despite these advantages, SERS-based biosensing presents significant analytical challenges, in particular for resolving mixtures of structurally related molecular species. The subtle spectral differences characteristic of closely related analytes^7^ require robust data analysis methods capable of recognizing individual components from the vibrational spectra of mixtures.^20^ It is common practice to use multivariate statistical analysis methods or machine learning models such as partial least squares discriminant analysis (PLS-DA), linear discriminant analysis (LDA) usually preceded by principal component analysis (PCA), random forest (RF), or support vector machine (SVM) for spectral analysis.^21,22^ Both RF and SVM algorithms have been extensively validated in SERS applications, each offering distinct analytical advantages.^23–27^ Random forest demonstrated superior robustness to outliers and noisy spectra while providing straightforward decision interpretation trees.^23,28^ Conversely, SVM generalizes well to new spectra through its usage of higher-dimensional feature spaces projected using a kernel function.^29^

Recently, data-driven deep learning techniques, mostly based on convolutional neural networks (CNN), have become increasingly popular for spectral analysis.^20,22,30,31^ Indeed, while two-dimensional (2D) CNNs are widely used for image analysis and object recognition, their one-dimensional (1D) counterparts are likewise particularly effective for 1D input data such as spectral data.^32–36^ These models are composed of several layers performing non-linear operations to convert the initial spectral features into task-specific abstract information. CNN models also feature convolutional layers to extract spatial correlation between adjacent spectral data points by applying a series of filters, thereby better exploiting the locally shared vibrational information contained in SERS spectra.^37^ Using CNNs for SERS analysis also benefits from the fact that it is relatively straightforward to generate artificial spectra using data augmentation methods, enabling model training on substantially expanded datasets. These models are thus of great interest for SERS analysis, as they can learn to recognize intricate spectral patterns combining several vibrational bands and often achieve higher performance than multivariate statistical analysis and conventional machine learning algorithms. Furthermore, compared to conventional approaches, CNN methods generally require minimal spectrum preprocessing and achieve more consistent performance across different preprocessing conditions, which facilitates their application to SERS spectrum analysis.^38–40^ In recent years, studies have shown the ability of CNN-type models combined with SERS spectroscopy to identify and differentiate single molecular species in biological samples.^41–43^ Conversely, the use of CNN to identify and quantify multiple molecular species simultaneously in individual samples, while being less common, can have far-reaching implications for clinical diagnostics.^44–47^ For example, definitive solid tumor characterization usually requires concurrent detection of multiple biomarkers prostate-specific antigen (PSA) and human epidermal growth factor receptor 2 (HER-2),^48,49^ where multiplexed analysis could significantly enhance diagnostic accuracy and clinical decision making.

Some of the reported attempts to detect multiple compounds from the vibrational spectra of mixtures use a distinct CNN model for the identification of each analyte.^44,50^ In this case, each model is trained to detect a single compound, and by combining several models it is possible to detect multiple compounds at the same time. However, the computational load for this approach scales with the number of target species, while each species is processed independently without sharing spectral information across models.^51,52^ Alternatively, a single CNN architecture with independent outputs can be used to determine the presence or absence of several molecules simultaneously.^39,53,54^ Furthermore, the above strategies can be used to enable simultaneous regression of multiple analytes. This is achieved by modifying the CNN output activation function (e.g., for a linear function) and the loss function (e.g., for a mean squared error function), thereby incorporating a quantitative aspect to the analysis.^53,55^ While CNN approaches hold great promise for the simultaneous detection of multiple molecular species in mixtures, experimental validation under physiologically relevant conditions remains limited. This includes assessing the model's ability to accurately differentiate between compounds in complex matrices^56^ or having similar spectral signatures or spectra with low signal to noise ratios.^57,58^ Incorporating such conditions at an early stage in the development of the analysis method is of the utmost importance to extend and promote the use of SERS in conjunction with CNN for simultaneous detection and classification of multiple species.

In the present work we implemented and tested a dual-stage analytical framework combining a multi-label CNN model to discriminate structurally similar analytes from SERS spectral data with a support vector regression (SVR) model for semi-quantitative analysis. Two short-chain fatty acids (SCFAs), a family of molecules being considered as markers for gut integrity and intestinal health,^59–62^ were chosen as SERS target species for this purpose, and measurements were performed with custom-fabricated gold nanostar-coated glass coverslips on SCFAs diluted either in water or in Dulbecco's Modified Eagle Medium (DMEM) cell culture medium. The multi-label CNN model is used to detect the presence or absence of the two analytes from the SERS spectra of the sample while the SVR model is used in parallel to determine the relative abundance of the species within the samples analyzed. Gradient-weighted class activation mapping (GradCAM), a post hoc method commonly used to visualize and understand the decisions made by CNNs,^63–65^ was used to analyze the multi-label CNN decision process for species identification. The novelty of this approach stems from the synergistic integration of a multi-label CNN model with an SVR model for enhanced classification and quantification of molecular mixtures in SERS. This dual-model framework operates in two complementary stages: The multi-label CNN first identifies and distinguishes complex mixtures from single-component samples, while the SVR model subsequently provides semi-quantitative analysis by determining the relative proportions of components within identified mixtures. This hierarchical approach enables more robust and informative characterization of molecular compositions, leading to enhanced analytical precision and more meaningful interpretation of SERS data.

## Experimental

## Materials and Methods

Reagents. Propionic acid (PA), valeric acid (VA), gold (III) chloride (HAuCl4, 99% purity), silver nitrate (AgNO3, 99.9995% purity), l-ascorbic acid (C6H8O6, 99% purity), and 2-(dimethylamino) ethanethiol hydrochloride (DMAET-HCl, 95% purity) were purchased from Sigma Aldrich. DMEM was purchased from Wisen Inc. (catalog no. 319-015), the detailed chemical composition of DMEM is provided in Table S1 (Supplemental Material). Anhydrous ethanol (99.9%) and Milli-Q water with a resistivity of 18.2 MΩ·cm were used for all experiments.

### Synthesis of Gold Nanostars

Gold nanostars (AuNSt) were synthesized using a seedless one-pot protocol described in a previous study,^66,67^ followed by an additional ligand exchange step with 2-(dimethylamino) ethanethiol (DMAET). Briefly, 1.44 mL of aqueous 10  mM chloroauric acid (HAuCl_4_) was added to 40 mL of water and vortexed for 10  s, followed by the addition of 50 µL of 10  mM aqueous silver nitrate (AgNO_3_) and vortexed for another 10  s and, finally, by the addition of 240 µL of aqueous 100  mM l-ascorbic acid and 20  s of vortex. The solution color changes from translucent yellow to greenish blue as HAuCl_4_ is reduced to form AuNSt.

Ligand exchange of l-ascorbic acid for capping agent DMAET was performed following a protocol adapted from literature.^68^ Given the greater affinity of DMAET for the gold surface compared to l-ascorbic acid, no pH modification or use of an additional compound was required for the ligand exchange to occur between these two compounds. Briefly, 28  mg of DMAET-HCl was added into 40 mL of a freshly synthesized AuNSt suspension and left to stir at 300  rpm for 3  h on an orbital shaker. The solution was then centrifuged two times at 2500 RCF for 25  min, resuspended in 40 mL of water, and stored at 4 °C in the dark. A redshift should be observed in the extinction spectrum of the AuNSt resulting from the refractive index change at the surface of the AuNSt (Figure S1, Supplemental Material). The optical extinction of the reaction media was monitored using ultraviolet–visible (UV–Vis) spectrophotometry (Agilent Cary-5000).

### Preparation of SERS Substrates

The SERS substrates were prepared by immobilizing AuNSt on commercial microscope coverslips using a meniscus evaporation-assisted electrostatic immobilization process used in a previous study.^67^ The coverslips were first cleaned by immersion in water and ultrasonicated for 5  min, followed by 10-min ultrasonication in anhydrous ethanol. The coverslips were then dried with a nitrogen stream and exposed to UV light for 10  min in a curing chamber (UVP CL-1000 Ultraviolet Crosslinker) to increase the number of available hydroxyl groups on the surface. To improve the electrostatic immobilization of cationic AuNSt on the coverslips, thin chambers were assembled onto the coverslips using poly- l-lysine-coated microscope slides and a Parafilm gasket. The thin chambers were filled with a concentrated AuNSt suspension, i.e., 1.5 mL of the initial AuNSt suspension centrifuged at 4000 relative centrifugal force (RCF) for 25  min and resuspended in 20 μL of water and dried under vacuum for 3  h. This process was repeated twice. As the AuNSt suspension evaporates, a meniscus is formed and moves across the chamber, producing a dense, uniform, and reproducible AuNSt film on the surface of the coverslip (Figure S1b, Supplemental Material). SERS substrates were then rinsed three times with water, dried with a nitrogen stream, and stored in the dark.

### Confocal Raman Microscope

A laboratory-built inverted confocal Raman microscope with a complementary brightfield imaging modality was used to measure SERS spectra (Figure S2, Supplemental Material). SERS substrates were scanned with a 21  mW helium neon laser (632.8  nm) excitation light source (4.6  mW at the sample plane) using a two-axis galvanometric laser scanning apparatus. An Olympus NiFluorite objective (40×, NA = 0.75) was used to focus the excitation light on the sample and to transfer the resulting Raman scattering, via a 50  µm core diameter multimode fiber (OZ Optics) serving as a confocal pinhole, to a compact spectrometer (Princeton Instrument’s Fergie) featuring a 1180 lines/mm grating blazed at 710  nm and a near-infrared (NIR)-enhanced charge-coupled device (CCD) detector cooled at −55 °C. The spectral range covered by the spectrometer was 650–780  nm (416–3100 cm^−1^). CCD images were acquired with a 1.0  s integration time, binned on-chip into 1 × 1024-pixel arrays and transferred to a PC using Princeton Instrument’s LightField software. A brightfield imaging modality composed of a 7  mW light-emitting diode (LED) centered at 525  nm and a monochrome complementary metal-oxide-semiconductor (CMOS) camera (Teledyne FLIR Model Blackfly S USB3) was used to visualize the SERS substrates and define the regions-of-interest (ROI) to be acquired during SERS experiments.

### SERS Measurements

The SCFA solutions were prepared in two different solvents (water and DMEM cell culture medium) and consisted of single-species solutions (VA or PA) at 100  µM as well as VA:PA mixtures in ratios of 4:1 (80:20  µM), 1:1 (50:50  µM), and 1:4 (20:80  µM). These concentrations are typical of physiological levels found in plasma and serum, but lower than levels found in the gut, which are in the millimolar range. It is important to mention that PA levels are generally higher than VA levels in plasma and serum, but this was not considered in this work.^60,69^ SERS substrates were immersed for 2  h in the SCFA solutions, gently rinsed with water, and dried with a nitrogen stream prior to SERS measurements. Additional substrates immersed in either solvent (without SCFA) following the same procedure were used to generate blank spectra.

**Figure 1.**
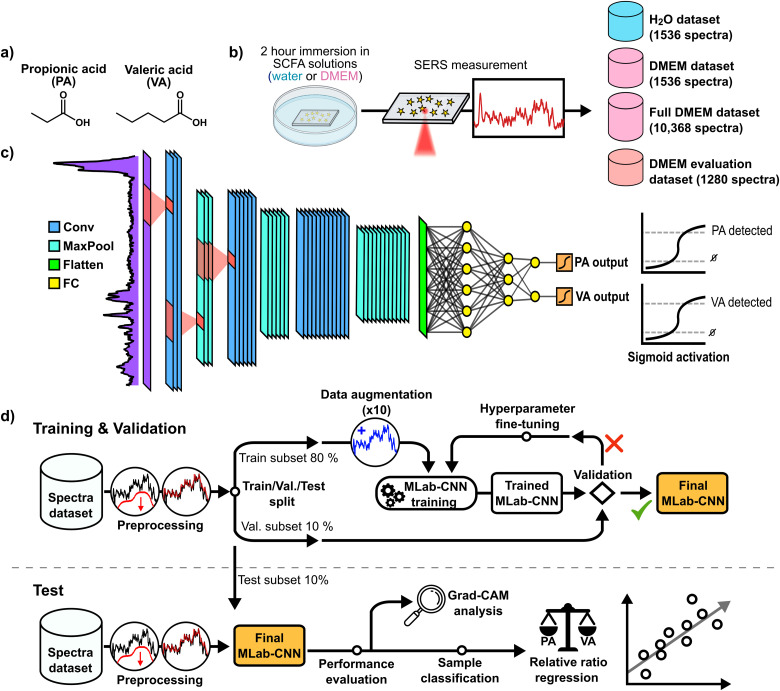
Experimental method used to perform the detection of SCFAs in SERS with deep learning. (a) Molecular structure of SCFAs propionic acid (PA) and valeric acid (VA). (b) Schematic summarizing the collection of SERS spectra datasets. (c) Architecture of the multi-label CNN used for detection of SCFAs. (d) Complete procedure to perform simultaneous multi-label deep learning-based detection of both SCFAs.

The number of substrates and spectra measured were different for the pure water- and DMEM-based samples: two substrates were used per sample for the former, including blanks, for a total of 12 substrates, whereas three substrates were used for DMEM-based samples for a total of 18 substrates. For each substrate, four to nine separate regions of 64 spectra (8 × 8 grids covering 110 × 110  µm) were measured. To increase variability, SERS measurements were taken at different times with multiple SCFA solutions at the same concentration. Measurement sequences were also randomized by switching between substrates between measurements to remove potential systematic bias. A total of 1536 and 10 368 spectra were measured, respectively, on water-based and DMEM-based SCFA samples and were grouped into two datasets called “H_2_O” and “full DMEM” datasets. To compare the behavior of the various models under study between the two sample types, a smaller spectral set (simply called “DMEM” dataset) was randomly selected from the full DMEM dataset to match the number of spectra measured in water. Finally, a separate dataset called “DMEM evaluation” dataset and intended for final model testing was measured on new DMEM samples using the same procedure as described above. This set included 1280 SERS spectra measured on five new samples, namely, four new VA:PA mixtures with ratios of 9:1 (90:10  µM), 65:35 (65:35  µM), 35:65 (35:65  µM), and 1:9 (10:90  µM), as well as a SCFA not present in the data, acetic acid (AA). A summary of the SERS measurement procedure and of the SERS spectra datasets collected is shown in Figure 1b).

## Data Processing and Learning Models

### Multi-Label Convolutional Neural Network Architecture and Training

The multi-label CNN was built with the Keras and Tensorflow packages in Python. Figure 1c depicts the structure of the model with its main building components.^70,71^ The first section of the model consists of three convolution layers each (8, 16, and 32 filters with kernel filters of size 5) followed by a maximum pooling layer. The second section of the model consists of three fully connected dense layers, with the latter serving as the output layer. The two model sections are joined by a Flatten layer. Rectified linear unit (ReLU) activation function is used for all layers except the output layer, where each classification output is assigned to an individual sigmoid activation function (see Supplemental Material for details on this function). Batch normalization layers are implemented right before ReLU activation functions to ensure faster and more stable multi-label CNN training convergence.

Multi-label CNN training was performed on a Nvidia Quadro P600 GPU using an Adam optimizer (learning rate = 1E-5) with a batch size of 132 and a binary cross-entropy function (see Supplemental Material for details on this function). The training was performed over 50 epochs. A 30% neuron dropout was applied in the two fully connected layers preceding the output layer during training to reduce the risk of overfitting and to increase generalizability on unseen SERS spectra (see Table S2 for the list of all hyperparameters values). Loss, precision, and recall were evaluated after each epoch on both the training and validation sets to ensure training convergence, and the model version that achieved the lowest validation loss was kept. Once the model had been trained, the analysis of precision and recall curves for different prediction thresholds was carried out for both model outputs to determine the optimal thresholds for PA and VA (Figure S3, Supplemental Material).

### SERS Dataset Handling and Labeling

The performance of machine learning models applied to the classification of SERS spectra was compared for measurements in a simple matrix (H_2_O dataset) and a complex matrix (DMEM dataset). Datasets from both media were split into training, validation, and testing with the proportions 80:10:10. To perform multi-label classification, each spectrum was associated with a two-class binary label indicating the presence of the two SCFAs. For instance, the label [0, 0] was assigned to the blank spectrum, labels [1–1] to mixtures of VA and PA. A separate testing dataset (DMEM evaluation dataset) comprising spectra from VA/PA mixtures in different ratios as well as a new SCFA (acetic acid) absent from the training datasets was prepared to assess the generalizability of the model to unpredictable sample compositions.

### Spectral Data Preprocessing and Augmentation

Data augmentation techniques were employed to generate synthetic spectra through systematic modification of existing spectral data, thereby expanding the training dataset for the CNN model. While these methods typically enhance model performance and mitigate overfitting risks,^72,73^ their application in Raman–SERS spectroscopy requires careful consideration due to the unique challenges associated with accurately replicating experimental spectral variations. The inherent sensitivity of SERS spectra to minute changes in substrate properties, sample composition, and environmental conditions poses significant challenges for data augmentation approaches. Inadequately designed augmentation strategies may fail to capture the true experimental variability, potentially creating a distribution shift between training and testing datasets that compromises model performance and limits generalizability to real-world applications.^74,75^

Several critical concerns must be addressed when implementing data augmentation for SERS data. Certain augmentation methods may inadvertently distort characteristic spectral features essential for accurate molecular identification, generate spectrally implausible data that violates fundamental Raman scattering principles, or introduce systematic biases by disproportionately emphasizing specific spectral regions while neglecting others of equal analytical importance.^75^ To ensure robust and reliable data augmentation, it is essential to integrate domain-specific expertise throughout the augmentation process. This includes validating that generated spectra remain physically meaningful and representative of genuine experimental conditions, as well as systematically evaluating each augmentation technique's contribution to improved model performance and enhanced generalizability across diverse experimental scenarios.^74,75^

Synthetic spectra were generated from the original training dataset using four distinct data augmentation strategies, each designed to simulate realistic experimental variations encountered in SERS measurements. The augmented spectra were subsequently integrated into the training dataset. The augmentation protocol comprised: (i) noise addition through both constant Gaussian noise and flicker noise (proportional to signal intensity) at a signal-to-noise ratio of 15  dB to replicate typical instrumental noise conditions; (ii) spectral shifting by 1–8 pixels along the Raman shift axis to account for potential calibration drift between measurements; (iii) linear baseline variation to simulate substrate-induced background fluctuations commonly observed in SERS experiments; and (iv) spectral mixing using linear combinations of two or three randomly selected spectra, with mixing coefficients sampled from a symmetric Dirichlet distribution (concentration parameter α = 0.4) to generate realistic mixture spectra.^67,76^

All augmentation parameters were systematically optimized through cross-validation to maximize performance enhancement while maintaining spectral authenticity. The concentration parameter and other hyperparameters were carefully tuned to avoid overly aggressive transformations that could lead to unrealistic spectral features and subsequent model underfitting on validation data. This comprehensive augmentation strategy resulted in a ten-fold expansion of the training dataset, increasing the available spectra from 8295 to 82 950 samples, thereby providing sufficient diversity for robust CNN model training while preserving the underlying spectroscopic characteristics essential for accurate molecular identification.

The most common methods for spectral preprocessing include smoothing, background correction, normalization and spectral truncation.^77,78^ Prior to data augmentation, all spectra were smoothed using a Savitzky–Golay filter (7-point, third-order polynomial, and 0˚ derivative), the central part (1800–2700 cm^−1^) where the Raman-silent region lies was removed, and intensity values were standardized with a Euclidean norm $\left(\sqrt{\sum I_{\;pixel}^{2}}=1\right)$ .

### Additional CNN-Based Models

Two other methods adapted from previous works and built around the same architecture as the multi-label CNN model were implemented and evaluated for SCFA detection. The first method, One-Sample CNN, involves two independent CNN models with a single detection output each.^44,50^ One model was trained for PA detection and the other for VA detection, and both models were applied at the same time to perform predictions on new spectra. The second method, called CNN regression, uses the architecture of the multi-label CNN model, but replaces the sigmoid outputs with dense linear ones. This model has been trained with binary soft labels with values corresponding to the molar ratio of SCFA, as well as mean squared error (MSE) loss function instead of binary cross-entropy loss, to do regression instead of classification.^53,55^

### Conventional Machine Learning Algorithms

The following multivariate machine learning algorithms were implemented and executed using the Scikit-learn Python library: (i) Partial least square discriminant analysis (PLS-DA) with 10 components, (ii) linear discriminant analysis (LDA) combined with principal component analysis (PCA) (60 components), (iii) a random forest (RF) model with 250 individual trees, and (iv) a support vector machine (SVM) model with a regularization parameter of 10 and a radial basis function (RBF) as the kernel.^79^

### Code Availability and Data Sharing

All raw and preprocessed spectra, as well as the deep learning models and functions programmed in Python, are freely available on the BoxSERS GitHub directory (https://github.com/ALebrun-108/BoxSERS).

## Results and Discussion

### Surface-Enhanced Raman Spectroscopy Spectra of Short-Chain Fatty Acids (SCFA) Mixtures

**Figure 2.**
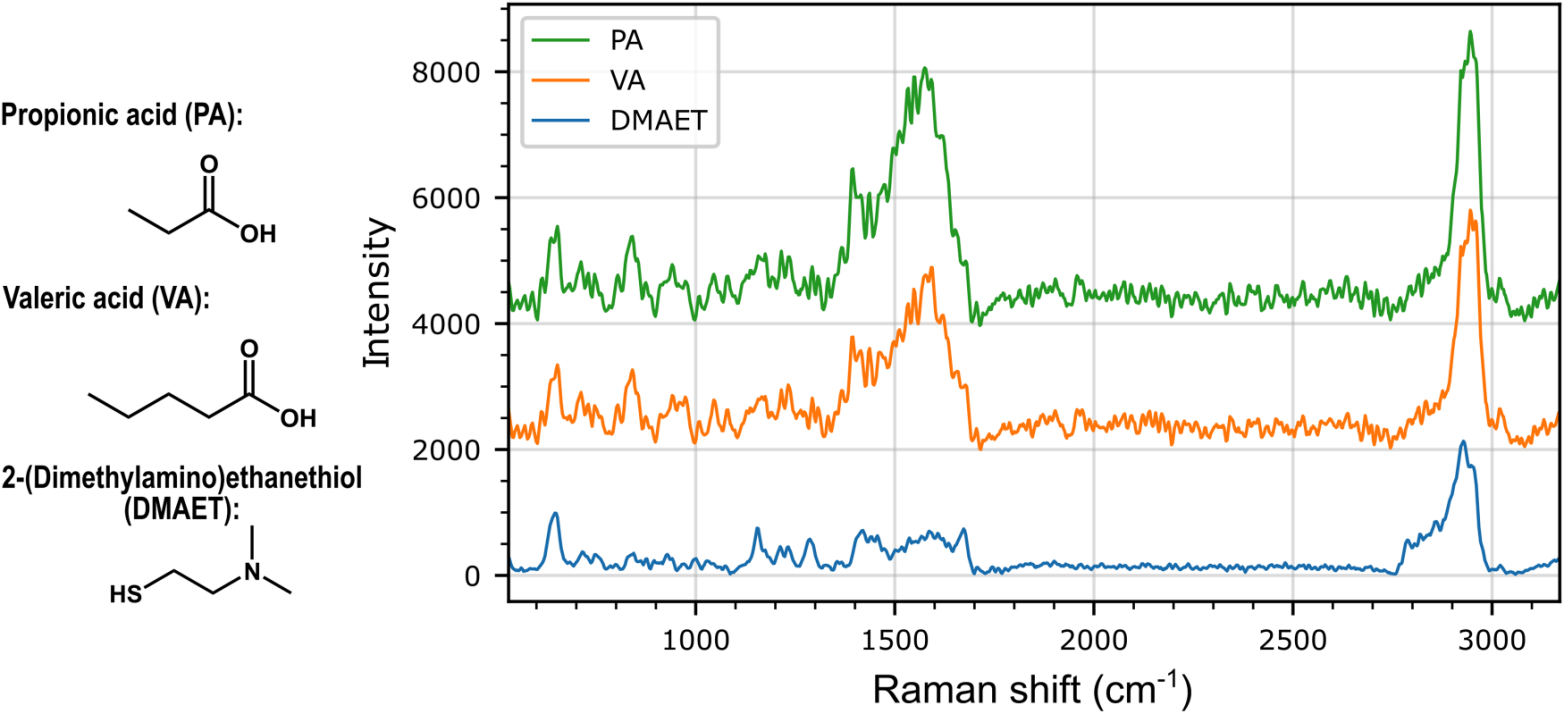
Average SERS spectra (*N* = 25 spectra) measured on 5  mM aqueous solutions of propionic acid (PA) and valeric acid (VA). The top two curves correspond to substrates immersed in each short-chain fatty acid solution for 2  h, while the lower curve was recorded from a substrate immersed in ultrapure water for the same duration and shows the background spectrum associated with the substrate surface ligand, 2-(dimethylamino)ethanethiol (DMAET). Spectra were measured with an integration time of 5  s, smoothed using a 7-point Savitzky–Golay filter and processed with an asymmetric least square baseline correction method.

Figure 2 shows average spectra (*N* = 25) measured on individual substrates after a 2-h immersion in concentrated (i.e., 5  mM) aqueous solutions of PA or VA, as well as on a blank substrate immersed in pure water only. These concentrations, higher than those used later in this work and usually found in biological samples (∼100  µM), were chosen to help with the visualization of spectral differences between individual SCFAs and the SERS substrate’s spectral background. The spectra for both SCFAs are significantly different from that for the blank substrate, in particular in the 1400–1700cm^–1^ region where several bands previously reported for SCFAs appear,^80,81^ namely the asymmetrical C–C stretching band centered at 975 cm^–1^, the two bands at 1044 and 1079 cm^–1^ corresponding to C–C stretching, C–O stretching and/or CH bending, the bands centered at 1394 and 1585 cm^–1^ associated respectively with symmetrical and asymmetrical O–C–O stretching, and finally, the band at 1535 cm^–1^ corresponding to CH_2_ bending. Another band outside the fingerprint region, centered at 2945 cm^–1^, is attributed to CH3 rocking and stretching. Sensitivity seems to be marginally higher for propionic acid, a fact that could be due to the greater affinity of PA for DMAET compared to VA; nevertheless, despite the high concentration used, the spectral features of both species are exceedingly similar, making the challenge posed by the discrimination of both species in mixtures an excellent opportunity to evaluate the performance of CNN classification.

**Figure 3.**
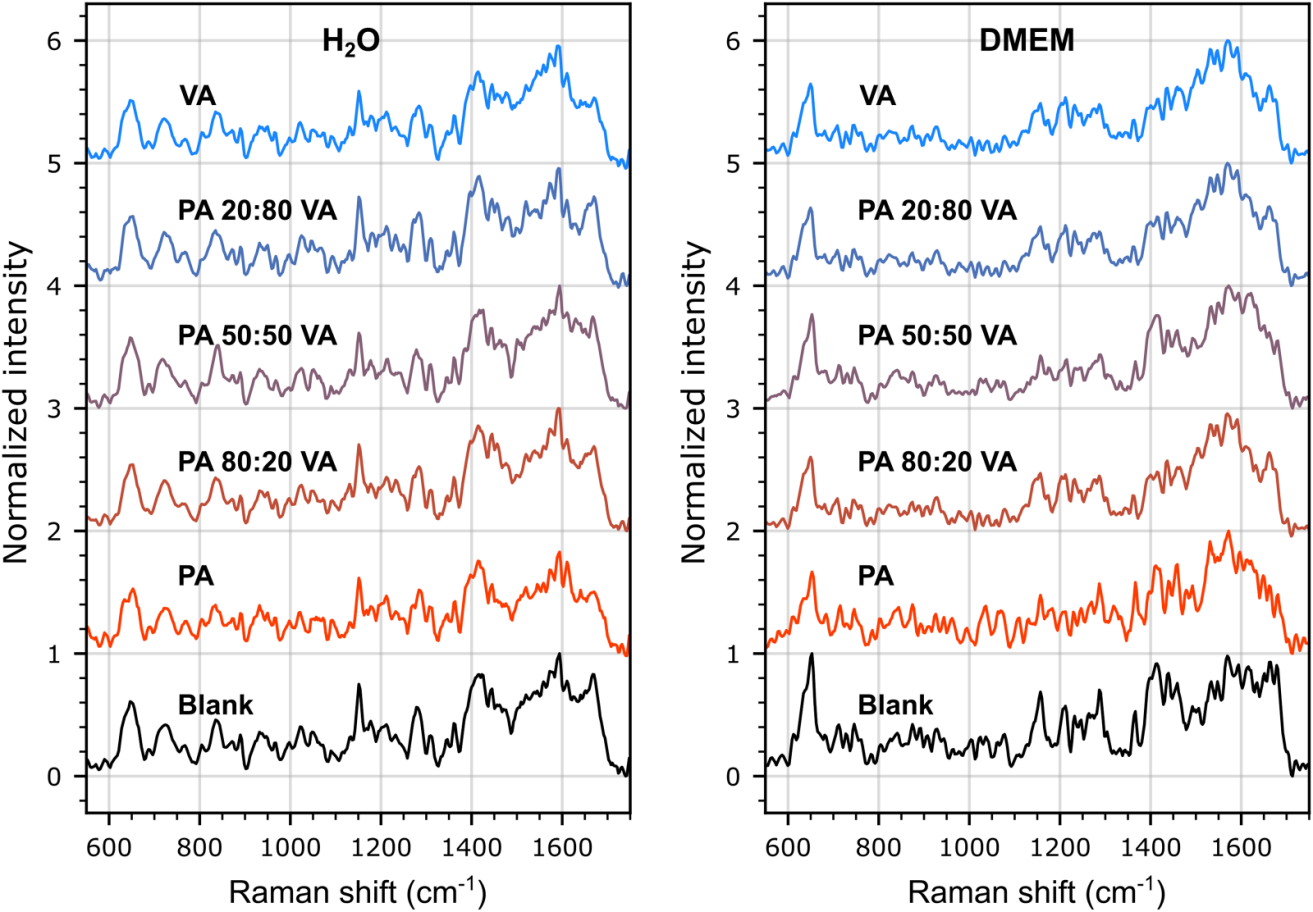
Average SERS spectra (*N* = 768 spectra/sample) of propionic acid (PA) and valeric acid (VA) solutions mixed in water (left panel) and in DMEM (right panel). The average spectra measured on blank SERS substrates are also presented. Each curve is labeled with the molar ratio on 100  µM of PA or VA in the solution. Spectra were measured with an integration time of 5  s, smoothed using a 7-point Savitzky–Golay filter and processed with an asymmetric least square baseline correction method.

The SERS spectra were also measured at lower concentration (100  µM combined) for PA and VA, alone in solution and as mixtures in varying molar ratios (80/20 to 20/80) dissolved either in pure water or in DMEM (Figure 3). A first observation is that the spectra from both SCFAs are exceedingly similar to that of the blank at this concentration level, and that transitioning from 100% PA to 100% VA has no readily discernable effect on the spectra that could be attributed to the dominance of one species over the other in the samples. A second observation is that the differences between the blank spectra for water and DMEM, the latter being rich in glucose, amino acids and electrolytes, are significantly greater than between the spectra for each SCFA, suggesting that any successful attempt at classifying these species using learning algorithms will requiring training the models with spectral databases recorded in sample matrices matched to those of the unknown samples.

### Multi-Label Detection of Short-Chain Fatty Acid Mixtures

The ability of the multi-label CNN and other conventional machine learning models to classify PA and VA in mixtures was evaluated using the H_2_O and DMEM datasets described in the experimental section. Precision and recall values were obtained from the models’ classification of the test dataset and F1 scores were subsequently calculated based on these metrics (see Supplemental Material for a discussion of the use of the F1 score). Average and standard deviation values were generated by running the entire procedure 10 times, each time reshuffling and dividing the dataset into new training, validation, and testing subsets. The F1 scores of the multi-label CNN model were compared with those for conventional non-convolutional machine learning methods and other CNN-type models (complete tabulated data, including precision and recall values, are provided in Tables S3 and S4, Supplemental Material).

**Figure 4.**
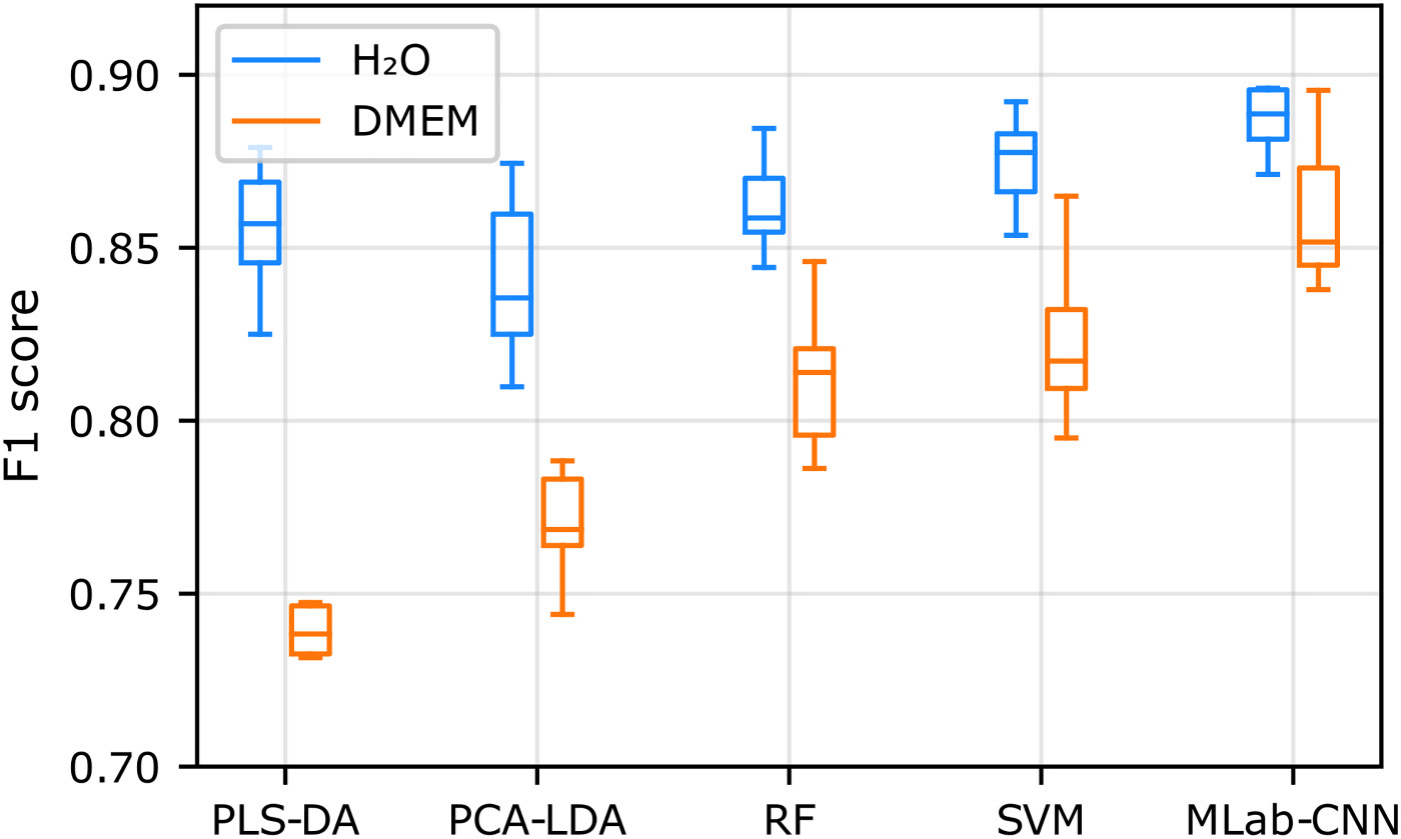
Box plot representation of the F1 scores obtained for SCFA detection with multi-label CNN and conventional machine learning classification models trained and tested on the H_2_O and DMEM.

Figure 4 shows that the F1 scores for the non-convolutional machine learning classification models and the multi-label CNN model are similar for SCFAs diluted in water (blue boxes) at physiological concentration levels, ranging from 0.84 ± 0.02 for PCA-LDA to 0.89 ± 0.02 for multi-label CNN. On the other hand, the performance of the non-convolutional algorithms was significantly impacted by the more complex DMEM sample matrix (yellow boxes), their average F1 score decreasing from 0.86 ± 0.01 in H_2_O to 0.79 ± 0.04 in DMEM, with SVM and RF performing slightly better, an observation in line with previously reported studies.^23–27^ In comparison, the multi-label CNN model was less affected by the sample matrix, with an F1 score decreasing slightly from 0.89 ± 0.02 in H_2_O to 0.86 ± 0.02 in DMEM. This higher generalizability of multi-label CNN may be related to the ability of CNNs to extract spectral features and transform them to a higher-level representation due to their deep architecture.^82^

**Figure 5.**
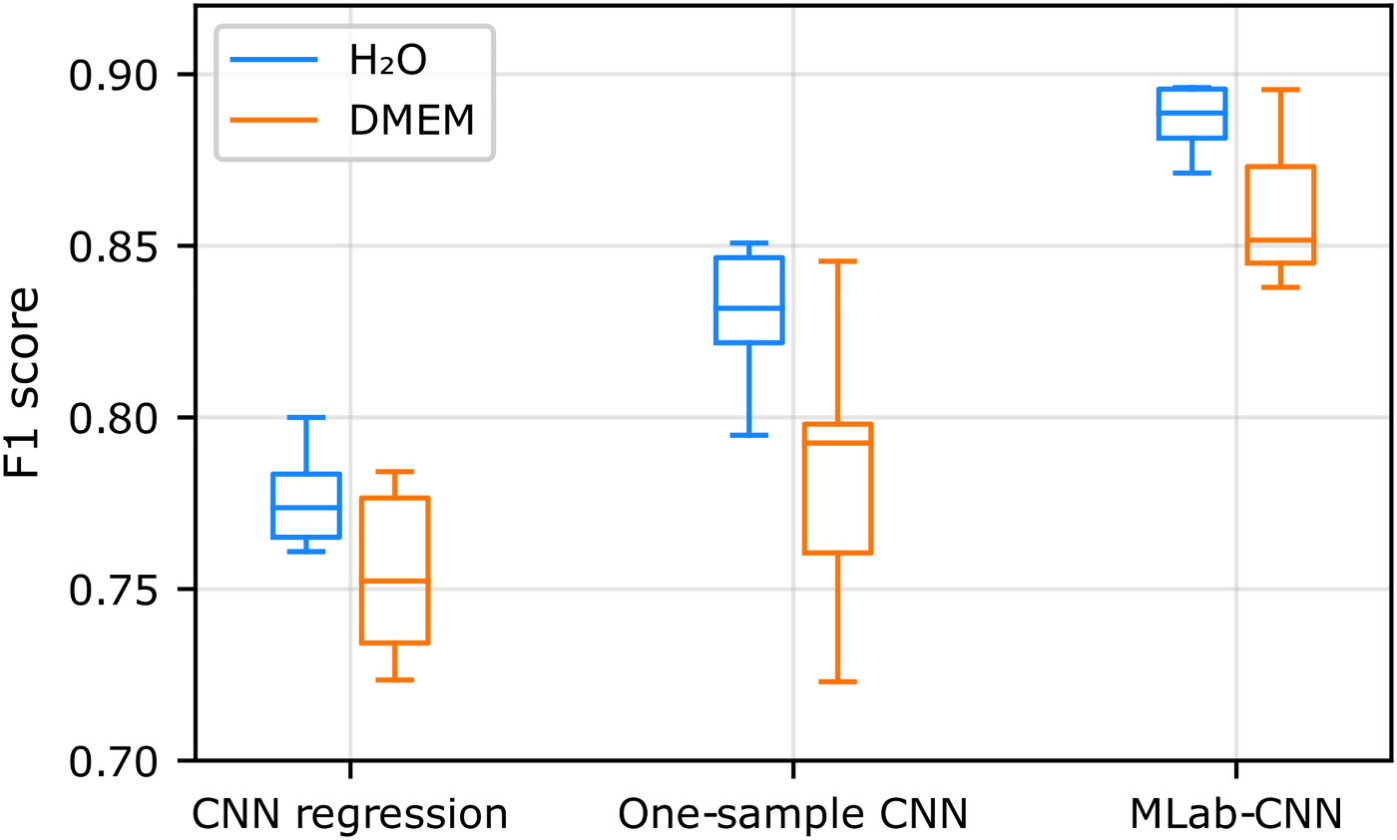
Box plot representation of the F1 scores obtained for SCFA detection with three CNN-based models when trained and tested on the H_2_O and DMEM datasets.

The multi-label CNN model achieved the highest F1 scores among the three CNN-based approaches tested in both aqueous and DMEM solutions (Figure 5), suggesting that it is particularly well suited for SERS measurements in complex matrices.

In particular, the sigmoid activation function used by multi-label CNN significantly improves multi-label detection performance compared to the linear activation function used by the Regression CNN model. This result was expected, as the latter is known to have a weaker ability to capture cross-species decision limits than sigmoid functions.^83^ Furthermore, unlike the one-sample CNN method where one model is trained to detect only one species without sharing information, the multi-label CNN approach is trained to detect both species simultaneously, enabling it to use shared and complementary information between the spectra of the two SCFAs.

This comparative study was conducted on a reduced subsample of spectra from the full DMEM set, adjusted in size to match the number of spectra measured in water, ensuring a valid comparison between the two matrices. When the study was repeated using the full DMEM set, all three CNN models, as well as the SVM and RF models, achieved higher F1 scores, with the multi-label CNN model once again obtaining the highest score. (Figure S4, Supplemental Material).

### Gradient-Weighted Class Activation Mapping (GradCAM) Interpretability Analysis

**Figure 6.**
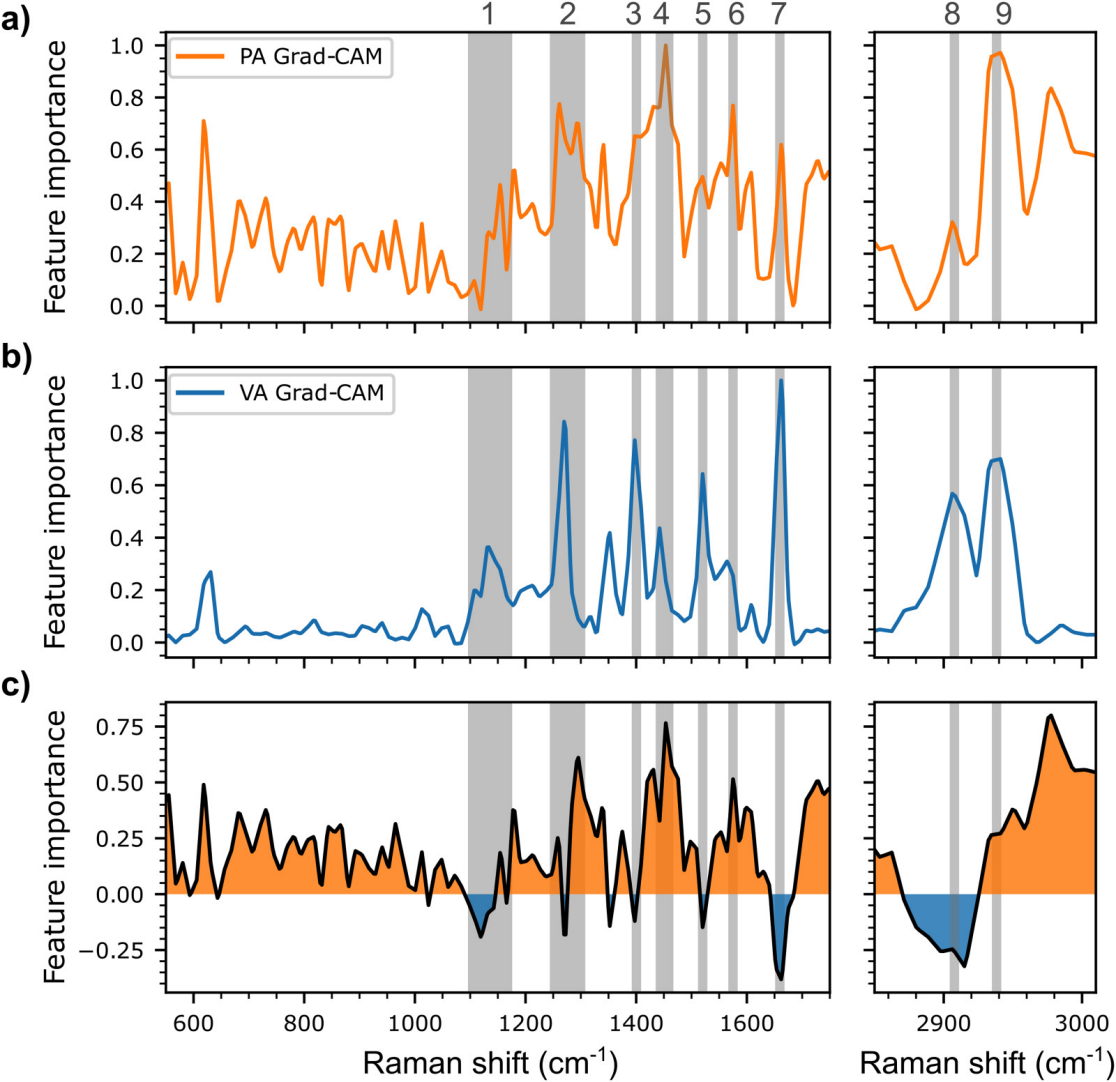
Spectrally distributed importance in the decision process of the multi-label CNN model, as determined using the GradCAM method, for the test spectra of (a) propionic acid (PA, in orange) and those of (b) valeric acid (VA, in blue). (c) GradCAM difference between PA and VA (PA minus VA) with the comparatively more important regions for PA (>0) in orange, and the comparatively more important regions for VA (< 0) in blue. Selected spectral bands or regions referenced in the analysis in the paper are highlighted with the light gray bands.

Post-hoc gradient-weighted class activation mapping (GradCAM) was applied to accurately predicted test spectra from SCFA mixtures to identify the key spectral bands or regions used by the multi-label CNN model to detect PA and VA. GradCAM analysis also enabled the evaluation of the SERS features guiding the predictions of the model (see the Supplemental Material for a detailed explanation of the GradCAM method). As shown in Figure 6, the bands used by the model include bands previously identified in Figure 2 along with additional bands that can be also attributed to functional groups of the two SCFAs. These include the region between 1100 and 1150 cm^–1^ (label 1), associated with CO stretching, OH and CH_2_ bending, the 1274–1304 cm^–1^ region (label 2) associated with CH2 twisting, the bands centered at 1394 (label 3) and 1585 cm^–1^ (label 6) associated with symmetrical and asymmetrical O–C–O stretching respectively, the band centered at 1450 cm^–1^ (label 4) associated to asymmetric CH_3_ bending, the band at 1535 cm^–1^ (label 5) corresponding to CH_2_ bending, the band centered near 1660 cm^–1^ associated to C=O (label 7), and finally the vibrational bands centered near 2900–2910 cm^–1^ (label 8) and 2940–2950 cm^–1^ (label 9) associated respectively to CH_2_ and CH_3_ stretching.^80^

Comparing the GradCAMs of the two SCFAs, it is possible to observe some particularly interesting differences, especially regarding the bands associated with the CH_2_ and CH_3_ groups. The band associated with the asymmetrical CH3 deformation (label 4) is more prominent in the case of PA, whereas the CH_2_ bending is more prominent for VA (label 5). Additionally, the gap between CH_2_ (label 8) and CH_3_ (label 9) stretching is smaller for VA than for PA. Both observations can be attributed to the difference in length of the aliphatic chain, and in the number of CH_2_ groups, of VA (*n* = 3) compared to PA (*n* = 1). Additionally, the more pronounced CH_3_ vibrational bands in PA may be explained by the shorter chain length, which increases the likelihood that the terminal CH_3_ group is closer to the surface of the SERS substrate.

GradCAM analysis underlines the subtle differences between the spectroscopic signatures used by the multi-label CNN model to detect PA and VA in mixture samples, thus revealing some of the vibrational mode essential to the model's decision-making process. In addition, it confirms that this model bases its prediction on valid spectral bands and improves the interpretability of the obtained detection results.

### SCFA Mixtures Classification with Multi-Label CNN and SCFA Ratio Assessment

**Figure 7.**
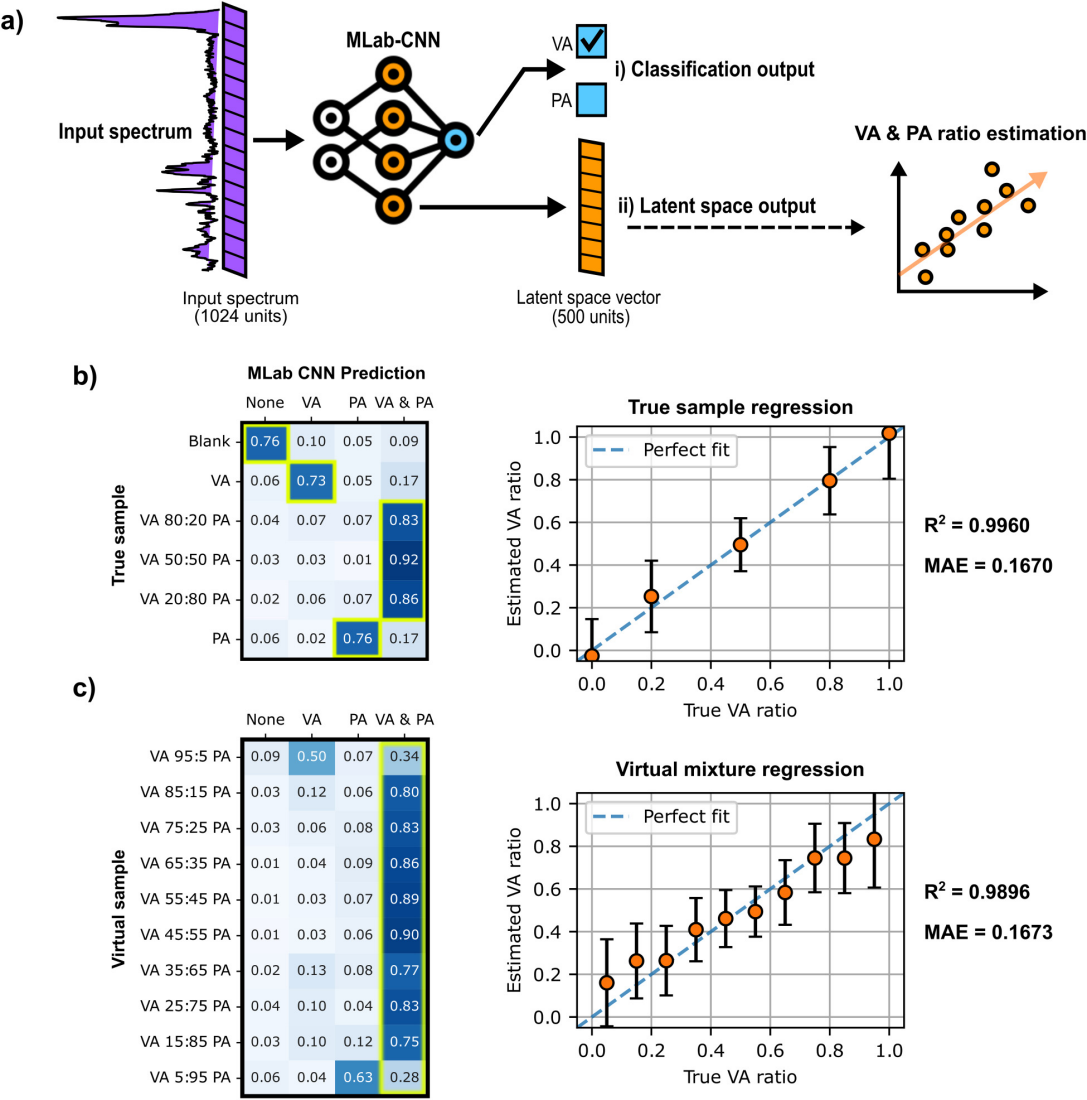
Multi-label CNN model classification and support vector regression (SVR) results for each SCFA sample. (a) The multi-label CNN model gives two different outputs: (i) the multi-label sigmoid predictions at the very end of the model used for classification and SCFA detection, and (ii) the latent vector extracted from the last dense layer preceding the output layer of the multi-label CNN model, which is used for subsequent regression analysis with the SVR. (b–c) Matrices of multi-label CNN predictions (columns) for each sample (rows) paired with a plot of SVR-predicted VA ratios and actual VA ratios for (b) five SCFA true samples and C) ten new SCFA virtual mixtures. The cells framed in yellow in the matrices correspond to the accurate classifications, and the dashed lines in regression plots shown in (b) and (c) correspond to ideal predictions (where the predicted values exactly match the actual values).

Having shown that the multi-label CNN model is able to identify and detect both SCFAs from different samples in aqueous and DMEM media, the next step was to assess the model's ability to correctly classify SCFA samples between the four possible classes: (i) Blank (no VA or PA), (ii) VA only, (iii) PA only, or (iv) a mixture of VA and PA. Furthermore, a support vector regression (SVR) model was used to infer the VA:PA ratio in samples identified as mixtures by multi-label CNN. Using an SVR model to estimate the ratio of VA to PA of the mixtures may provide more information on the spectra predicted as mixtures. SVR model training was performed on spectra from the original DMEM training dataset, using as input the latent space vector derived from the last dense layer of the multi-label CNN model (Figure 7a). The labels used for the regression correspond to the relative molar fraction of VA in the samples, e.g., the spectrum of a mixture with a VA: PA ratio of 4:1 (80:20  µM) was labeled as 0.8. The approach was validated through a five-fold cross-validation that compared two regression models and different input types in term of mean absolute error (MAE) (Table S5, Supplemental Material).

Figure 7b (left panel) presents the classification results achieved by the multi-label CNN model for the six sample mixtures in the study. The distribution of the model's prediction for each sample is summarized in a matrix that provides transparent insight on the model's performance. multi-label CNN gives consistent classification results for all six samples, assigning most spectra to the correct class with an accuracy of ( 75±2 ) % for the blank and single-component SCFA samples (rows 1, 2, and 6), and ( 87±5 ) % for SCFA mixtures (rows 3, 4, and 5), confirming that the model can recognize the presence or absence of each SCFA, both individually and in mixtures. A significant proportion of true spectra of single SCFAs (17%) (rows 2 and 6) were misclassified as mixtures of VA and PA (column 4), meaning that the multi-label CNN model tends to predict the presence of an absent SCFA when the other SCFA is actually present. This potential sensitivity issue can be attributed to the complexity of SCFA spectra and the similarity of the two species, which can favor imprecise simultaneous detection of both species. However, given that the error is much smaller in terms of individual detection, and that the use of a complementary regression method is likely to provide additional information to compensate for this issue, this situation is judged as non-problematic. Also, in cases where it would be desirable to reduce the number of spectra wrongly classified as mixtures by the multi-label CNN, it is possible to adjust the detection thresholds of the model’s two sigmoid outputs to increase precision and reduce the number of false positives.

As shown in Figure 7b (true sample regression plot), the SVR model succeeded in predicting VA:PA ratios consistent with expected ratios in the six samples tested, achieving an R2 of 0.9963 and a mean absolute error (MAE) of 0.1670. This plot also reveals that the error is more significant for single-component samples than for mixtures, underestimating the ratios contained in single SCFA samples. These results thus support the validity of using a semi-quantitative analysis alongside classification to estimate molar ratios in the sample analyzed.

To extend the multi-label CNN + SVR combined workflow to a larger number of SCFA mixtures, and to evaluate how the model performs on mixture ratios not included in the model training, 10 additional SCFA mixtures were evaluated. These new mixtures, shown in Figure 7c, were artificially generated from the same DMEM test set used to generate the results shown in Figure 7b, through linear combination of various sample spectra (250 spectra were generated for each mixture). As shown in the classification matrix (Figure 7c), excluding the mixtures closest to single-component samples (VA:PA ratios of 95:5  µM and 5:95  µM), the multi-label CNN model successfully classifies mixtures spectra with an accuracy of ( 80±7 )%. This demonstrates the capacity of the multi-label CNN to generalize to new SCFA mixtures that were absent during training, supporting its broader applicability. Reduced classification performance is obtained for the mixtures closest to single-component samples (first and last rows), where a higher proportion of spectra were misclassified as individual VA or PA samples (50% and 63%, respectively). These results seem to reflect the detection limits of the classification method, where the relative concentration of a species in the mixture is sufficiently low to result in misclassification by multi-label CNN.

Finally, as shown by the virtual regression plot (Figure 7c), the SVR model succeeded in predicting the expected ratios for these 10 mixtures with R2 and MAE values of 0.9896 and 0.1673, respectively, close to the values obtained with true sample regression (Figure 7b). Therefore, the classification performed by the multi-label CNN model and the regression performed by the SVR model proved that they could be successfully applied in a complementary way on different SCFA samples.

### Multi-Label CNN Test on SERS Spectra of an Unknown Molecule and Additional Mixtures

As a last step, the generalizability of the combined multi-label CNN + SVR model approach was tested on an out-of-distribution dataset with mixtures and single species samples of PA and VA, as well as a SCFA absent from the training set, acetic acid (AA). This last species was selected due to its documented abundance in the gastrointestinal tract as well as to a molecular structure closer to PA than VA. To address the experimental variability inherent to SERS measurements, a relatively small number (300) of spectra, measured at the same time as this new dataset and comprising only single-species SCFA spectra or blanks, was used to re-train and benchmark the multi-label CNN model for this out-of-distribution dataset.

**Figure 8.**
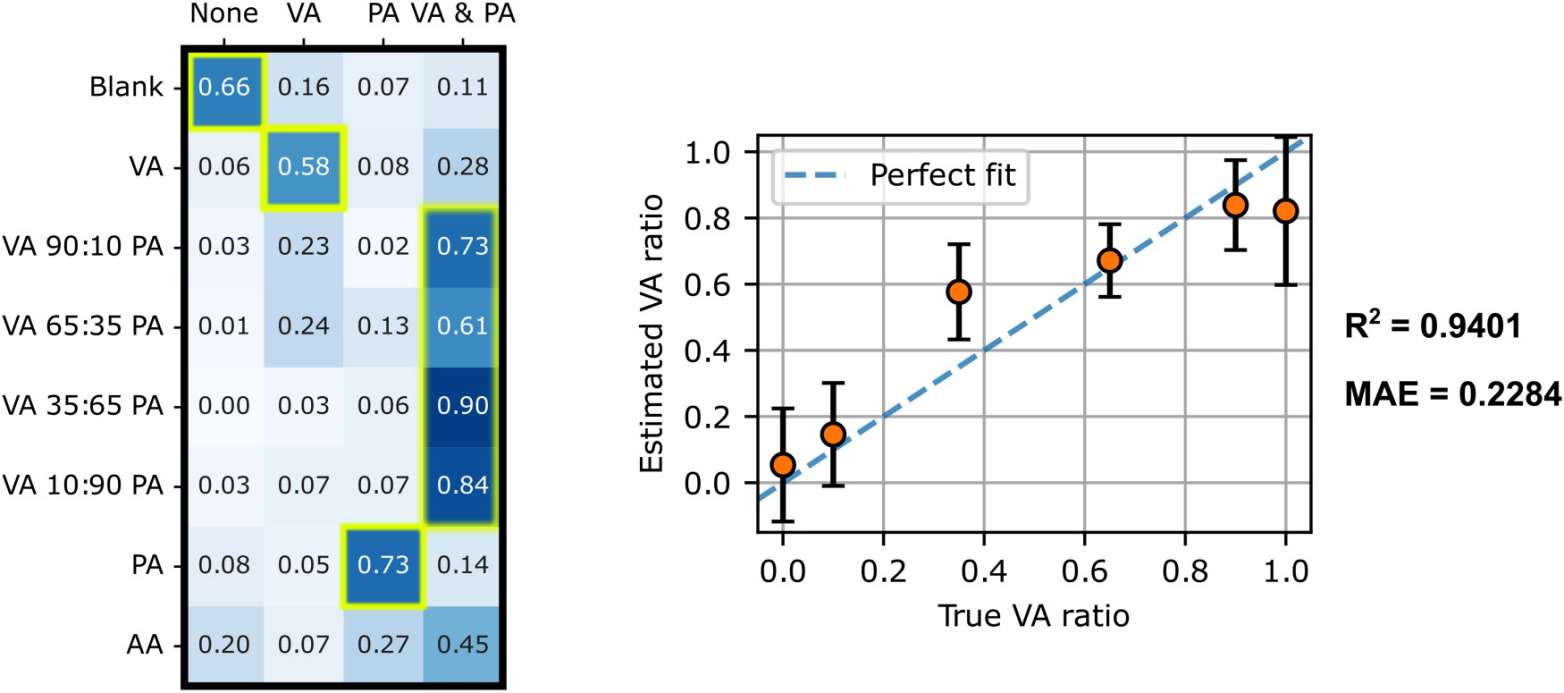
Multi-label CNN model classification and SVR results for unseen SCFA sample. Left: Matrix of multi-label CNN predictions (columns) for each sample (rows) paired with a plot of SVR-predicted VA ratios and actual VA ratios. The matrix cells framed in yellow correspond to the accurate classifications and the dashed lines in the regression plot (right) correspond to ideal predictions (where the predicted value exactly matches the actual values).

As shown in the matrix in Figure 8 (left), (66±8)% of spectra were accurately classified as the blank or single-component VA or PA samples (rows 1, 2, and 7), and (77±9)% for the new mixtures (rows 3–6). These results are statistically equivalent to those obtained previously for the other SCFA samples tested, which attests to the generalizability of the multi-label CNN for classifying SCFA mixtures. Regarding the SVR results (Figure 8, right), an R2 of 0.9401 and a MAE of 0.2284 were achieved on this set of spectra. These results show that the approach maintains a correlation between the actual and estimated VA ratios, confirming the generalizability of the method.

With regard to the classification of acetic acid (AA), unknown to the multi-label CNN model, 45% of spectra were classified as PA + VA mixtures, 27% as PA and 20% as neither. The reason why the model predictions lean toward mixtures or PA alone can be explained by the greater similarity between AA and PA molecular structures. Compared to the other SCFA samples evaluated in this study, the percentage of spectra classified in the “none” class is relatively high. This outcome suggests that applying a threshold on spectra classified as “none” (no VA or PA detected) could be used to rule out samples that are considered non-conclusive. These results thus give valuable insight on how the multi-label CNN handles confounding non-targeted species, a situation most likely to occur in real biological samples.

## Conclusion

The present study provides a new method for the classification of complex mixtures in SERS, based on a multi-label CNN classification model and SVR regression, which was validated on binary mixtures of SCFA, PA, and VA, in both simple aqueous solutions and complex DMEM culture media. Using this method, both SCFAs were successfully detected in varied ratios, with a precision of (90±1) % and a sensitivity (recall) of (91±1) %. By comparing the performance of this model with other approaches, the multi-label CNN proved to be not only more efficient at detecting both SCFAs, but also more robust with regards to complex sample matrices. A GradCAM analysis revealed that valid and spectrum-relevant variations in the SERS spectra are used by the model to detect and distinguish the two SCFA species in the different samples. The method also demonstrated robust performance in accurately classifying six different mixture ratios and ten new artificially generated samples. The integration of CNN classification with SVR enabled the accurate estimation of VA:PA ratios, achieving correlation coefficients (R2) of 0.9963 and 0.9896 for the respective sample sets. These results establish a strong foundation for the application of multi-label CNN-SVR frameworks to complex biological mixture analysis in SERS.

Future research directions will focus on expanding the analytical scope through systematic increases in molecular diversity while incorporating realistic environmental perturbations such as temperature fluctuations, pH variations, and matrix effects that characterize authentic biological samples. This progression toward greater complexity will require substantial methodological refinements, including enhanced CNN architectures with increased model capacity and significantly expanded training datasets, likely requiring an order-of-magnitude increase in spectral measurements to maintain robust performance. The successful demonstration of this proof-of-concept approach provides a clear pathway for advancing SERS-based mixture analysis toward clinically relevant applications, where the ability to simultaneously identify and quantify multiple biomarkers in complex biological matrices represents a critical analytical capability.

## Supplemental Material

sj-docx-1-asp-10.1177_00037028251377474 - Supplemental material for Surface-Enhanced Raman Spectroscopy Semi-Quantitative Molecular Profiling with a Convolutional Neural Network Supplemental material, sj-docx-1-asp-10.1177_00037028251377474 for Surface-Enhanced Raman Spectroscopy Semi-Quantitative Molecular Profiling with a Convolutional Neural Network by Alexis Lebrun, Flavie Lavoie-Cardinal and Denis Boudreau in Applied Spectroscopy
