# Supplementary material for: Surface-Enhanced Raman Spectroscopy Semi-Quantitative Molecular Profiling with a Convolutional Neural Network
Source: Appl Spectrosc. 2025 Aug 31;80(1):35–50. doi: 10.1177/00037028251377474 (PMC12717294; doi:10.1177/00037028251377474)
Supplement: sj-docx-1-asp-10.1177_00037028251377474 - Supplemental material for Surface-Enhanced Raman Spectroscopy Semi-Quantitative Molecular Profiling with a Convolutional Neural Network [file sj-docx-1-asp-10.1177_00037028251377474.docx]

**Supplemental Material**

**Surface-Enhanced Raman Spectroscopy Semi-Quantitative Molecular Profiling with a Convolutional Neural Network**

Alexis Lebrun^1,2,3,5^, Flavie Lavoie-Cardinal^2,3,4^, Denis Boudreau^1,5^*

^1^Centre d'optique, Photonique et Laser (COPL), Université Laval, Quebec, Canada

^2^Centre de Recherche CERVO, Université Laval, Quebec, Canada

^3^Institut Intelligence et Données, Université Laval, Quebec, Canada

^4^Département de Psychiatrie et Neurosciences, Université Laval, Quebec, Canada

^5^Département de Chimie, Université Laval, Quebec, Canada

*Corresponding author email: denis.boudreau@chm.ulaval.ca


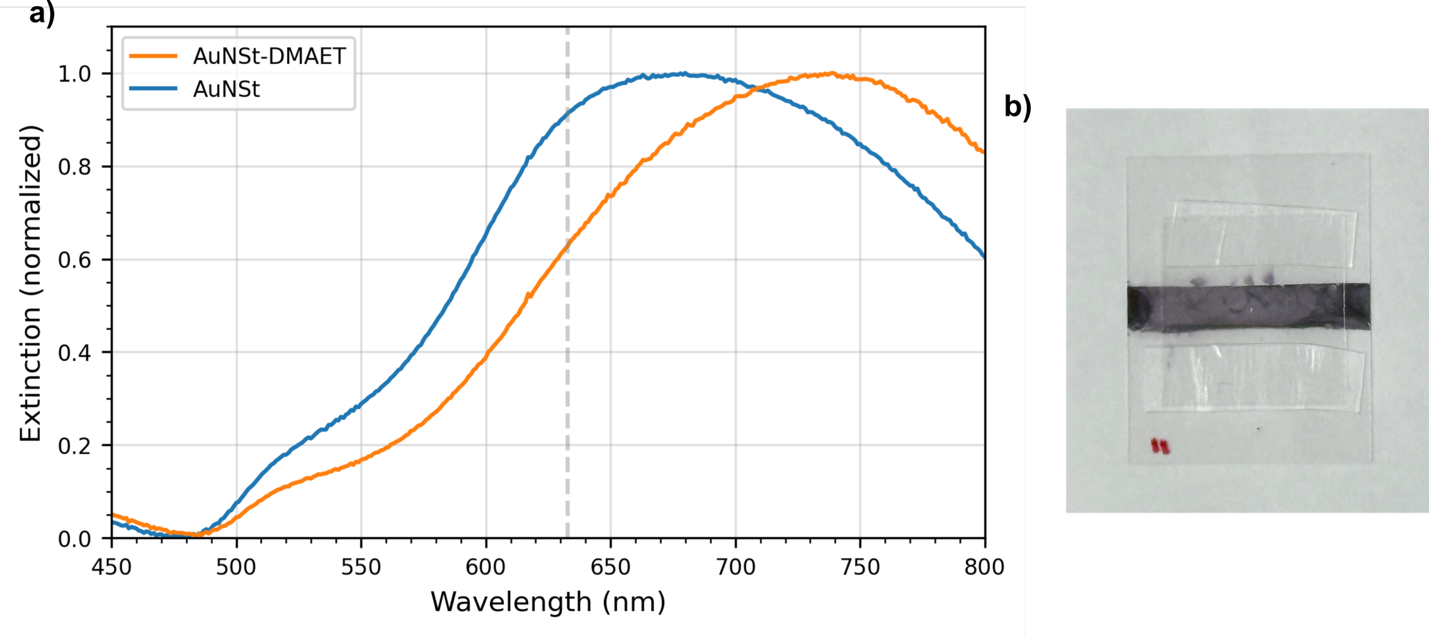


**Figure S1.** (a) UV-Vis extinction spectrum of gold nanostars (AuNSt) in solution before and after ligand exchange with capping agent 2-(dimethylamino)ethanethiol (DMAET). Ligand exchange with DMAET produces a redshift of 60 nm (679 to 739 nm). The dashed gray line corresponds to the Raman excitation wavelength of 632.8 nm. (b) Image of a SERS substrate after immobilization of AuNSt-DMAET on a glass coverslip.


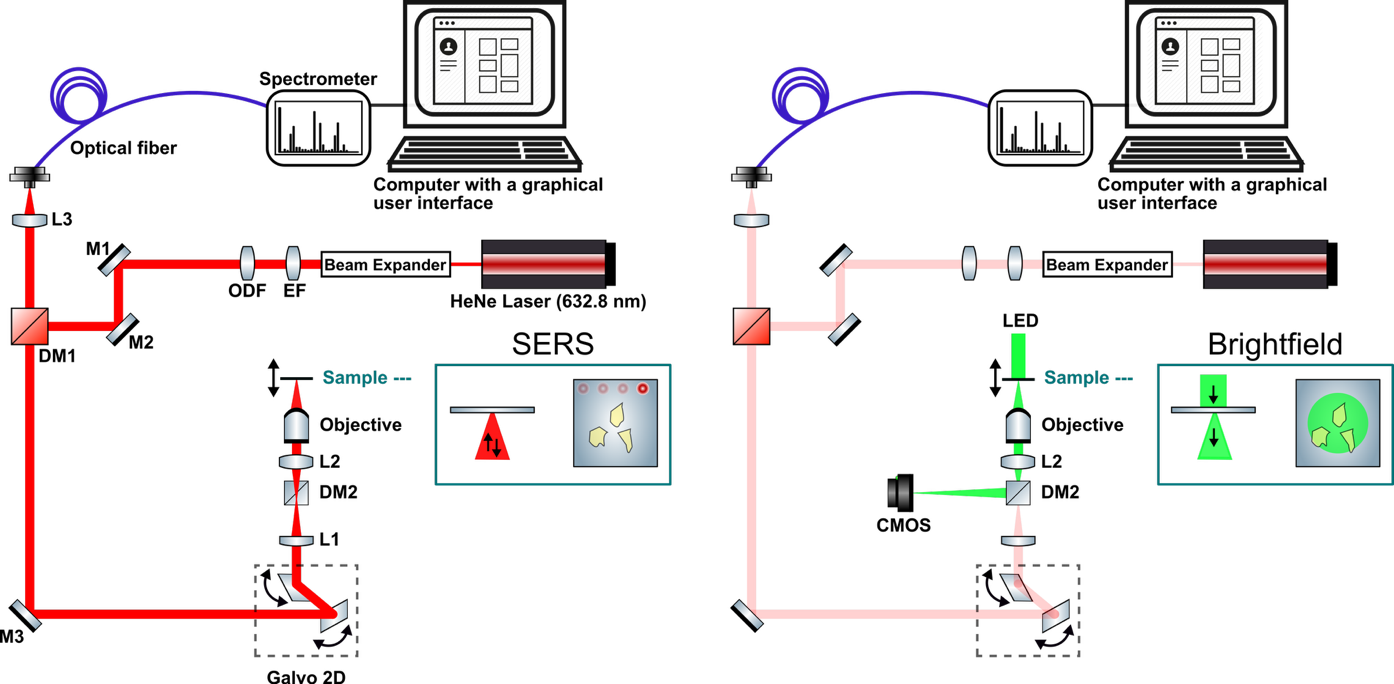


**Figure S2.** Diagram of the dual-modality microscope setup with the confocal Raman imaging modality (left, red path) and the bright-field imaging modality (right, green path). EF: excitation filter; ODF: optical density filter; M: mirror; DM: dichroic mirror; L: lens; and CMOS: complementary metal-oxide-semiconductor.

**Table S1.** Detailed composition of Dulbecco's Modified Eagle Medium (DMEM) (taken from the supplier's website at https://www.wisentbioproducts.com/fr/product/319-015-cs-dmem-1x-with-4-5-g-l-glucose-l-glutamine-w-o-sodium-pyruvate-10-x-500ml/).

| **Catalog no.** | **319-015 1X, liquid DMEM** |
| --- | --- |
| Inorganic salts | mg/L |
| CaCl_2_ (anhydrous) | 200.00 |
| Fe(NO_3_)_3_·9H_2_O | 0.10 |
| KCl | 400.00 |
| MgSO_4_ (anhydrous) | 97.70 |
| NaCl | 6400.00 |
| NaH_2_PO_4_·H_2_O | 125.00 |
| NaHCO_3_ | 3700.00 |
| Amino acids | |
| L-Arginine·HCl | 84.00 |
| L-Cystine·2HCl | 62.57 |
| L-Glutamine | 584.00 |
| Glycine | 30.00 |
| L-Histidine·HCl·H_2_O | 42.00 |
| L-Isoleucine | 104.80 |
| L-Leucine | 104.80 |
| L-Lysine·HCl | 146.20 |
| L-Methionine | 30.00 |
| L-Phenylalanine | 66.00 |
| L-Serine | 42.00 |
| L-Threonine | 95.20 |
| L-Tryptophan | 16.00 |
| L-Tyrosine·2Na·2H_2_O | 103.79 |
| L-Valine | 94.00 |
| Vitamins | |
| D-Calcium pantothenate | 4.00 |
| Choline chloride | 4.00 |
| Folic acid | 4.00 |
| i-Inositol | 7.20 |
| Nicotinamide | 4.00 |
| Pyroxidine·HCl | 4.00 |
| Riboflavin | 0.40 |
| Thiamine·HCl | 4.00 |
| Other | |
| D-Glucose | 4500.00 |
| Phenol red, Na | 15.00 |
| Sodium pyruvate | − |
| Specifications | |
| pH (after buffer) | 7.2 ± 0.2 |
| Osmolality (mOsm/kg) | 335 ± 30 |

**Computational Details**

**Table S2.** List of all hyperparameters used this work to train the multi-label convolutional neural network (multi-label CNN) to detect propionic and valeric acid from SERS spectra.

| Hyperparameters | Value |
| --- | --- |
| Epoch | 50 epochs |
| Batch size | 132 spectra |
| Learning rate | 1E-5 |
| Optimizer | Adam |
| Kernel size | (1×5) |
| Padding | Zero padding |
| Stride | 1 |
| Loss | Binary loss |
| Batch Normalization | True |
| Dropout rate | 0.3 |

*
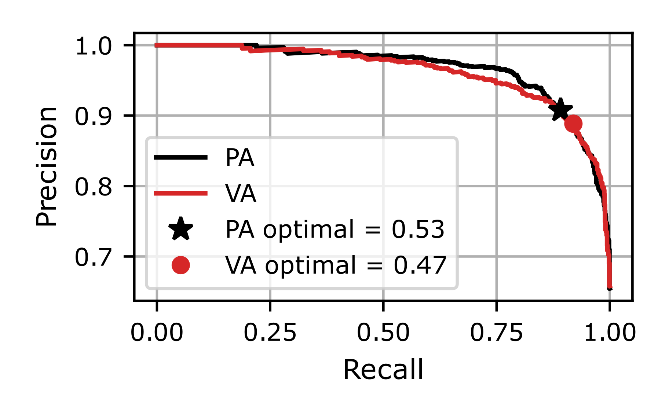
*

**Figure S3.** Precision and recall curves for the propionic acid (PA) and valeric acid (VA) CNN outputs at different thresholds using validation spectra (*N* = 1786). The threshold values that maximize the F1 score are shown in both curves.

*Model Performance Metrics for Multi-Label Detection*

*Precisi*on corresponds respectively to the fraction of true positives (TP) among all positives detected and recall corresponds to the fraction of true positives detected among all true positives. The F1 score is the harmonic mean of these two values, as shown here:

$Precision= \frac{TP}{TP+FP}, Recall=\frac{TP}{TP+FN}, F1= \frac{2\cdot Precision\cdot Recall}{Precision+Recall}$

where FP represents false positives and FN false negatives. These three metrics were first evaluated separately for PA and VA, and then macro-averaged for the three metrics, i.e., giving equal weight to each SCFA, according to the following equation:

${Metric}_{macro}=\frac{{Metric}_{PA}+{Metric}_{VA}}{2}$

In this work, improving precision at the expense of lower recall would increase confidence in the predicted PA and VA spectra, but would also reduce the sensitivity of the models to detect SCFAs. Conversely, increasing recall at the expense of precision would ensure fewer undetected PA and VA predictions, but it would result in a higher number of false positives, i.e., PA incorrectly predicted as VA or the opposite. Therefore, unless the application leans more towards precision or recall, it is usually a good practice to use the F1 score to compare models, as it balances precision and recall. For this reason, hyperparameters and the threshold values were optimized to maximize the F1 score.

*Sigmoid Cross-Entropy Loss Calculations*

For the multi-label CNN model, a sigmoid activation function ($\sigma$) was applied independently to the outputs of the CNN associated with each class:

$\hat{y}_{i}=\sigma\left( z_{i} \right)=\frac{1}{1+e^{-z_{i}}}$(S1)

Equation 1 gives the predicted probability $\hat{y}_{i}$ based on the neural network’s raw output $z_{i}$ for the $i$th class. During the neural network training, binary cross entropy, also known as sigmoid cross-entropy loss, is used to measure the difference between the model’s predicted probabilities $\hat{y}_{i}$ and the expected class output.^1^

*Gradient-Weighted Class Activation Mapping (GradCam) Calculations*

GradCam is a technique used to visualize and understand the decisions made by convolutional neural networks (CNNs). This post hoc method can be used to increase explainability and transparency of a CNN model, without compromising its architecture or performance.^2^ In SERS spectroscopy, this technique has been used to highlight the regions in the vibrational spectra, and the molecular groups involved, that have the greatest influence on a model's decisions.^3–6^

To produce a class-discriminative localization map $L_{\text{GradCAM}}^{c}$ that highlights important regions of a spectrum about a predicted *p* or selected class $c$, the GradCam technique backpropagates the gradient calculated at the model output down to the last convolution layer. Any layer of the CNN can be used for GradCam, but it is usually the last convolutional layers that are chosen, as they offer the best compromise between high-level semantics and detailed spatial information. The gradient is computed from the partial derivative of the model’s raw output $z_{i}$ for the $i$th class (before the sigmoid), with respect to the $k$th feature map activations $A_{p}^{k}$ of the last convolutional layer. The gradient is then global-average-pooled over the spectrum length dimension $p$ at the convolutional layer of interest to compute the neuron importance weights $\alpha_{k}^{c}$ as in the following equation,

$\alpha_{k}^{c}=\overset{\begin{aligned} \text{Global average} \\ \text{pooling} \end{aligned}}{\overbrace{\frac{1}{Z}\sum_{p}}}\underset{\text{Gradients}}{\underbrace{\frac{\partial z^{c}}{\partial A_{p}^{k}}}}$ (S2)

where Z is the total length of the feature map. The weight $\alpha_{k}^{c}$ represents the importance of the feature map $k$ in relation to a target class $c$. Applying a rectified linear unit function ($\mathrm{ReLU}(x)=max(0, x)$) on the weighted linear combination of the activations $A_{p}^{k}$ with the computed weights $\alpha_{k}^{c}$ results in the class-discriminative localization map $L_{\text{GradCam}}^{c}$ as shown in the next equation:

$L_{\text{GradCam}}^{c}=ReLU\underset{\text{linear combination}}{\underbrace{\left( \sum_{k} \alpha_{k}^{c}A^{k} \right)}}$(S3)

To compare $L_{\text{GradCam}}^{c}$ properly and draw conclusions on the spectral characteristics that have the greatest influence on the model's decisions, the GradCam maps (length equal to the dimension at the last convolutional layer) were interpolated to match the length of the spectra and normalized to improve comparison.

**Results and Discussion**

**Table S3.** Averaged precision, recall and F1 values (± standard error) achieved with different conventional machine learning models for multi-label classification of two short-chain fatty acids (SCFAs) in aqueous and in Dulbecco's Modified Eagle Medium (DMEM) solutions. Model tested were partial least squares discriminant analysis (PLS-DA), linear discriminant analysis combined with principal component analysis (PCA-LDA), random forest (RF), and support vector machine with a radial basis function kernel. Average values (macro type) were calculated by training and testing each model 10 times on different train/test spectra splits.

| Model |  | Precision  (H_2_O) | Recall  (H_2_O) | F1  (H_2_O) |  | Precision (DMEM) | Recall  (DMEM) | F1  (DMEM) |
| --- | --- | --- | --- | --- | --- | --- | --- | --- |
| PLS-DA |  | $0.81\pm0.04$ | $\boldsymbol{0}.\boldsymbol{90}\pm\boldsymbol{0}.\boldsymbol{02}$ | $0.86\pm0.02$ |  | $0.74\pm0.04$ | $0.72\pm0.02$ | $0.74\pm0.03$ |
| PCA-LDA |  | $0.83\pm0.03$ | $0.85\pm0.02$ | $0.84\pm0.02$ |  | $0.74\pm0.03$ | $0.79\pm0.03$ | $0.77\pm0.02$ |
| RF |  | $0.86\pm0.02$ | $0.86\pm0.02$ | $0.86\pm0.01$ |  | $\boldsymbol{0}.\boldsymbol{80}\pm\boldsymbol{0}.\boldsymbol{02}$ | $0.81\pm0.02$ | $\boldsymbol{0}.\boldsymbol{82}\pm\boldsymbol{0}.\boldsymbol{03}$ |
| SVM |  | $\boldsymbol{0}.\boldsymbol{87}\pm\boldsymbol{0}.\boldsymbol{01}$ | $0.89\pm0.01$ | $\boldsymbol{0}.\boldsymbol{87}\pm\boldsymbol{0}.\boldsymbol{02}$ |  | $0.77\pm0.04$ | $0.86\pm0.02$ | $\boldsymbol{0}.\boldsymbol{82}\pm\boldsymbol{0}.\boldsymbol{03}$ |

**Table S4.** Averaged precision, recall and F1 values (± standard error) achieved with different CNN-based approaches for multi-label classification of two short-chain fatty acids (SCFAs) in aqueous and DMEM solutions. Average values (macro type) were calculated by training and testing each model 10 times on different train/test spectra splits.

| Model |  | Precision  (H_2_O) | Recall  (H_2_O) | F1  (H_2_O) |  | Precision (DMEM) | Recall  (DMEM) | F1  (DMEM) |
| --- | --- | --- | --- | --- | --- | --- | --- | --- |
| Regression CNN |  | $0.82\pm0.03$ | $0.72\pm0.01$ | $0.78\pm0.01$ |  | $0.76\pm0.01$ | $0.76\pm0.06$ | $0.75\pm0.02$ |
| One-sample CNN |  | $0.85\pm0.03$ | $0.83\pm0.03$ | $0.84\pm0.03$ |  | $0.80\pm0.04$ | $0.77\pm0.04$ | $0.78\pm0.04$ |
| Multi-label CNN |  | $\boldsymbol{0}.\boldsymbol{88}\pm\boldsymbol{0}.\boldsymbol{04}$ | $\boldsymbol{0}.\boldsymbol{91}\pm\boldsymbol{0}.\boldsymbol{03}$ | $\boldsymbol{0}.\boldsymbol{89}\pm\boldsymbol{0}.\boldsymbol{02}$ |  | $0.84\pm0.02$ | $0.88\pm0.01$ | $0.86\pm0.02$ |
| Regression CNN  (full DMEM set) |  | $-$ | $-$ | $-$ |  | $0.80\pm0.01$ | $0.78\pm0.02$ | $0.79\pm0.01$ |
| One-sample CNN (full DMEM set) |  | $-$ | $-$ | $-$ |  | $0.86\pm0.01$ | $0.85\pm0.01$ | $0.85\pm0.01$ |
| multi-label CNN  (full DMEM set) |  | $-$ | $-$ | $-$ |  | $\boldsymbol{0}.\boldsymbol{90}\pm\boldsymbol{0}.\boldsymbol{01}$ | $\boldsymbol{0}.\boldsymbol{91}\pm\boldsymbol{0}.\boldsymbol{01}$ | $\boldsymbol{0}.\boldsymbol{91}\pm\boldsymbol{0}.\boldsymbol{01}$ |


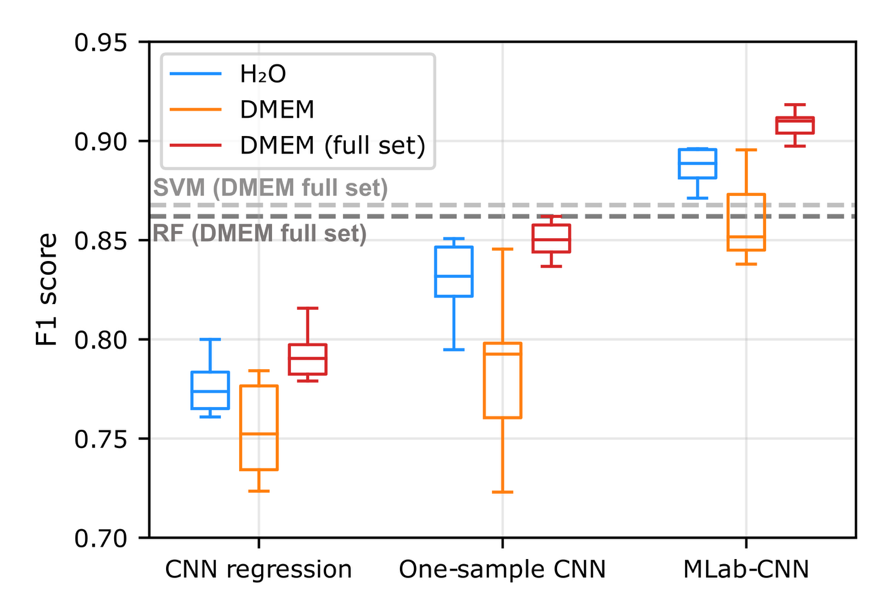


**Figure S4.** Box plot representation of the F1 scores obtained for SCFA detection with three CNN-based models when trained and tested on the H_2_O, DMEM and Full DMEM datasets. SVM and RF model top F1 score obtained on Full DMEM dataset are also shown.

**Table S5.** Averaged mean absolute error (MAE) achieved with a linear regression (LR) and a support vector regression (SVR) models for different inputs. Average values were calculated based on five different train/test spectra splits.

| Model | Input |  |  | MAE |
| --- | --- | --- | --- | --- |
| LR | Input spectrum |  |  | $0.29\pm0.02$ |
|  | Last conv. layer |  |  | $0.29\pm0.02$ |
|  | First dense layer |  |  | $0.25\pm0.02$ |
|  | Last dense layer |  |  | $0.21\pm0.02$ |
| SVR | Input spectrum |  |  | $0.25\pm0.02$ |
|  | Last Conv. layer |  |  | $0.24\pm0.02$ |
|  | First dense layer |  |  | $0.21\pm0.02$ |
|  | Last dense layer |  |  | $0.18\pm0.03$ |

**References**

1. X. Ke, J. Zou, Y. Niu. “End-to-End Automatic Image Annotation Based on Deep CNN and Multi-Label Data Augmentation”. IEEE Trans. Multimedia. 2019. 21(8): 2093–2106. 10.1109/TMM.2019.2895511

2. R.R. Selvaraju, M. Cogswell, A. Das, R. Vedantam, et al. “GradCAM: Visual Explanations from Deep Networks via Gradient-Based Localization”. ArXiv. 2016. 10.48550/arXiv.1610.02391

3. C.-C. Xiong, S.-S. Zhu, D.-H. Yan, Y.-D. Yao, et al. “Rapid and Precise Detection of Cancers via Label-Free SERS and Deep Learning”. Anal. Bioanal. Chem. 2023. 415(17): 3449–3462. 10.1007/s00216-023-04730-7

4. G. Shi, H. Wu, S. Luo, X. Lu, et al. “1D Gradient-Weighted Class Activation Mapping, Visualizing Decision Process of Convolutional Neural Network-Based Models in Spectroscopy Analysis”. Anal. Chem. 2023. 95(26): 9959–9966. 10.1021/acs.analchem.3c01101

5. P. de Carvalho Gomes, A. Crossman, E. Massey, J.J. Stanley Rickard, P.G. Oppenheimer. “Real-Time Validation of Surface-Enhanced Raman Scattering Substrates via Convolutional Neural Network Algorithm”. Inform. Med. Unlocked. 2022. 33: 101076. 10.1016/j.imu.2022.101076

6. A.M. Fuentes, K. Milligan, M. Wiebe, A. Narayan, et al. “Stratification of Tumour Cell Radiation Response and Metabolic Signatures Visualization with Raman Spectroscopy and Explainable Convolutional Neural Network”. Analyst. 2024. 149(5): 1645–1657. 10.1039/d3an01797d
